# Radiocarbon in otoliths of tropical marine fishes: Reference Δ^14^C chronology for north Caribbean waters

**DOI:** 10.1371/journal.pone.0251442

**Published:** 2021-05-12

**Authors:** Virginia R. Shervette, Katherine E. Overly, Jesús M. Rivera Hernández

**Affiliations:** 1 Fish/Fisheries Conservation Lab, University of South Carolina Aiken, Aiken, SC, United States of America; 2 University of South Carolina, Marine Sciences, Columbia, SC, United States of America; 3 Riverside Technology for the NOAA National Marine Fisheries Service, Panama City Laboratory, Panama City, Florida, United States of America; Institute of Marine Research, NORWAY

## Abstract

Reef fishes support important fisheries throughout the Caribbean, but a combination of factors in the tropics makes otolith microstructure difficult to interpret for age estimation. Therefore, validation of ageing methods, via application of Δ^14^C is a major research priority. Utilizing known-age otolith material from north Caribbean fishes, we determined that a distinct regional Δ^14^C chronology exists, differing from coral-based chronologies compiled for ageing validation from a wide-ranging area of the Atlantic and from an otolith-based chronology from the Gulf of Mexico. Our north Caribbean Δ^14^C chronology established a decline series with narrow prediction intervals that proved successful in ageing validation of three economically important reef fish species. In examining why our north Caribbean Δ^14^C chronology differed from some of the coral-based Δ^14^C data reported from the region, we determined differences among study objectives and research design impact Δ^14^C temporal relationships. This resulted in establishing the first of three important considerations relevant to applying Δ^14^C chronologies for ageing validation: 1) evaluation of the applicability of original goal/objectives and study design of potential Δ^14^C reference studies. Next, we determined differences between our Δ^14^C chronology and those from Florida and the Gulf of Mexico were explained by differences in regional patterns of oceanic upwelling, resulting in the second consideration for future validation work: 2) evaluation of the applicability of Δ^14^C reference data to the region/location where fish samples were obtained. Lastly, we emphasize the application of our north Caribbean Δ^14^C chronology should be limited to ageing validation studies of fishes from this region known to inhabit shallow water coral habitat as juveniles. Thus, we note the final consideration to strengthen findings of future age validation studies: 3) use of Δ^14^C analysis for age validation should be limited to species whose juvenile habitat is known to reflect the regional Δ^14^C reference chronology.

## Introduction

Reef fishes provide many ecosystem services in temperate to tropical marine systems around the globe. These include subsistence, recreational, and commercial fisheries, in addition to nutrient cycling, trophic transfer of organic carbon through the food web, and habitat engineering, among others. Over the past 30 years, Caribbean islands have experienced declines in commercial catches of marine fishes [1, 2]. Longer-term fishing pressure in multi-species fisheries results in shifts in the fish communities and impacts the population demography within populations of targeted species [3–6]. Despite their intrinsic and extrinsic value, even heavily targeted reef fishes in many global regions, including the U.S. Caribbean, are considered to be data-limited/data-deficient, often lacking estimates of basic life-history parameters, such as population age structure, longevity, growth, and natural mortality. These parameters are clearly fundamental to modern integrated stock assessments, but they are also utilized in data-limited assessment approaches when data on the magnitude or age composition of removals or fishery-independent indices of abundance are lacking [7, 8].

Time, or age, is the fundamental parameter to estimate population dynamics, with otolith analysis often providing age estimates of bony fishes via reads of alternating translucent and opaque zones in otolith thin sections. However, lower environmental variability among seasons or complex life histories (e.g., habitat transitions, sex change, long life) can make interpretation of otolith microstructure difficult for age estimation in tropical fishes. There is a premium, therefore, on verification or validation of age estimation for tropical reef fishes.

Bomb radiocarbon is a useful tool that has been applied to the validation of age estimates in fishes. The ^14^C was introduced into the atmosphere as a result of nuclear bomb testing in the 1950s to around 1970 [9]. Subsequently, ^14^C dissolved into ocean CO_2_ and was incorporated into the aragonite (biogenic CaCO_3_) skeletons of hermatypic corals [10–12], carbonate-based shells of mollusks [13, 14], and the aragonite structures of fishes [15]. The incorporation of bomb-produced radiocarbon is reported as Δ^14^C in reference to a pre-nuclear proliferation standard [16]. The temporal marine record of radiocarbon increase and decline at low latitudes has been documented for multiple oceanic regions through the analysis of Δ^14^C in annual accretions of biogenic CaCO_3_ in hermatypic corals [11, 12].

The time-specific Δ^14^C coral records provide regional reference chronologies that are used to evaluate fish age estimates through comparison of fish Δ^14^C measured in otolith core material that formed during early life. Fish otoliths are composed principally of aragonite and are metabolically inert once formed, with ^14^C incorporated into their structure from both dissolved inorganic carbon (DIC) and food sources [17]. Analyzing the Δ^14^C contained within the otolith core (birth year) and then comparing that value to the predicted value from a regional Δ^14^C coral time series enables one to test the accuracy of age estimates [15]. The bomb radiocarbon chronometer historically was applied to validate age estimation for long-lived (>30 y) reef fishes that had birth years in the 1950s and 1960s [18] during the period of rapid rise in coral Δ^14^C, but more recently the linear decline in coral Δ^14^C since the 1970s has been applied to validate bony fish age estimates of younger and more recently collected fishes [19, 20].

**Fig 1:**
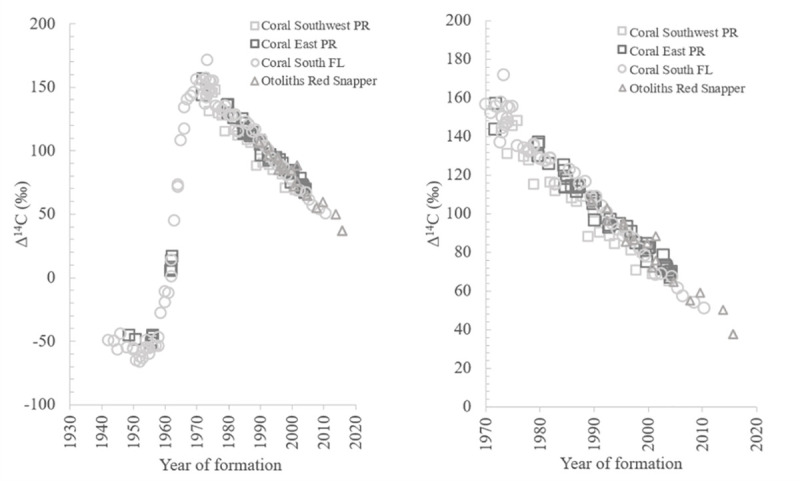
Scatterplots of Δ^14^C values versus year of formation for reference chronologies. Reference chronologies are from southwest PR coral, east PR coral, south FL coral, and GOM known-age red snapper otoliths. Left plot includes time range of 1940–2020 which covers the end of the pre-bomb ^14^C, bomb ^14^C rise, and bomb ^14^C decline periods. Right plot highlights the decline period from 1970 to 2020.

For north Caribbean and adjacent waters of the western Atlantic, four main ^14^C chronologies exist (Fig 1): one from Florida Keys [10], one from the east coast of Puerto Rico [21], one from the southwest coast of Puerto Rico [22], and one from Gulf of Mexico (GOM) [20]. The Florida Keys reference chronology was obtained from annual growth bands of the coral *Orbicella annularis* for 1800–1974 [10] and extended to 2010 [23]. On the eastern coast of Puerto Rico, Δ^14^C was reported from annual bands of *O*. *annularis* from 1950–2004. The southwest coast of Puerto Rico’s reference chronology was also established from growth bands of *O*. *annularis* and covers the years 1751–2004 [22]. A fourth reference chronology consists of Δ^14^C of known-age red snapper *Lutjanus campechanus* from the Gulf of Mexico [20].

The overall goal of this study was to develop a Δ^14^C decline relationship for the north Caribbean using known-age otoliths of red hind *Epinephelus guttatus* that can be used to validate ageing methods for north Caribbean fishes. Our specific objectives were: 1) to analyze the Δ^14^C of known-age reference red hind otoliths and examine whether the linear relationship between year of formation and Δ^14^C was significantly different from the Caribbean and GOM decline series; 2) to use the new region-specific north Caribbean bomb radiocarbon chronometer to validate age estimates of red hind by analyzing Δ^14^C in otolith cores with birth years from the past three decades; 3) to test the applicability of the red hind Δ^14^C chronometer for other bony fishes from the north Caribbean; and 4) to identify important considerations to strengthen the findings of future age validation studies utilizing Δ^14^C reference chronologies. Ultimately, this study provides a scientifically rigorous model of utilizing known-age fish otoliths to establish Δ^14^C chronometers for other regions throughout the wider Caribbean and elsewhere for which coral Δ^14^C time series are unavailable or may differ from reference Δ^14^C collections.

## Methods

This study was carried out in strict accordance with the recommendations in the Guide for the Care and Use of Laboratory Animals of the National Institutes of Health. The protocol was approved by the University of South Carolina Aiken Institutional Animal Care and Use Committee (Protocol Number: 053012-BIO-04).

### Sampling region and study species

**Fig 2:**
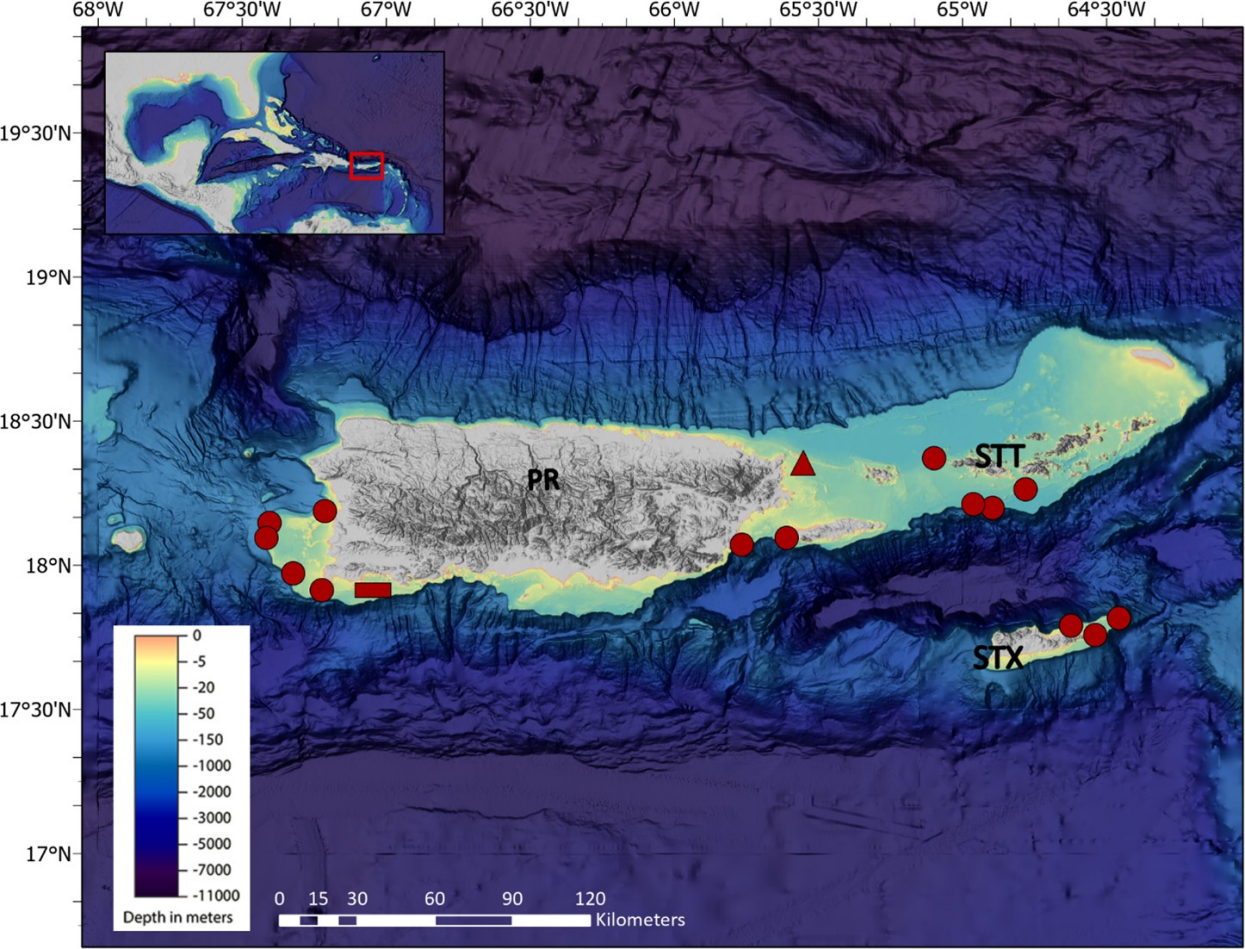
Sampling region in the north Caribbean. Map indicates sample locations for red hind (circles) and the locations of Δ^14^C coral reference chronologies in southwest waters (rectangle) and eastern waters (triangle) of Puerto Rico. The map layer used to generate this figure is from NOAA National Centers for Environmental Information and provided without restriction by the U.S. Government.

The U.S. Caribbean, located in the western part of the Caribbean archipelago, is ~1770 km southeast of the Florida Keys, and includes Puerto Rico (PR) and the U.S. Virgin Islands (USVI; Fig 2). USVI is part of the Virgin Islands chain and is located ~ 80 km east of PR (Fig 2). The major islands of USVI are St. Thomas (STT), St. John (STJ), and St. Croix (STX). STT and STJ are surrounded by the Atlantic Ocean to the north and the Caribbean Sea to the south (Fig 2). STX, located south of STT and STJ, is entirely surrounded by the Caribbean Sea. Caribbean currents characterized by large cyclonic and anticyclonic gyres, flows 1000 km south of the U.S. Caribbean islands. Average speed of the Caribbean current ranges from 0.5–1 knots and its strength is influenced by shifts in the position of the Inter-tropical Convergence Zone (ITCZ) [24]. The ITCZ shifts are the main cause for seasonal changes in precipitation with a dry season from late winter to spring when the ITCZ is near the equator and a wet season (summer-fall) when the ITCZ has shifted to its most northerly position. Sea surface temperature ranges from a February-March minimum of 25°C to an August-September maximum of 29°C [24].

Red hind is a moderately long-lived, shallow-water grouper reef fish species that occurs throughout the Caribbean and the western Atlantic in tropical and subtropical waters in association with shallow water coral reef habitat throughout its life. It is a protogynous hermaphrodite that within the study region forms monthly spawning aggregations cued by the full moon starting as early as December until as late as April [25–27] at specific aggregation sites near the shelf edge characterized by complex structure in the form of dense scleractinian coral [28]. One study from over 30 years ago investigated age and growth for red hind in the north Caribbean [29] and documented that opaque zone formation in sagittal otoliths occurs annually from February-July. The maximum reported age for red hind from this region is 18 y [29]. We selected red hind otoliths for documenting the regional pattern of Δ^14^C for two main reasons: 1) the past work on this species involved archiving otolith samples from across the U.S. Caribbean which provided us access to otoliths collected over multiple decades; and 2) the documentation concerning peak spawning month and months that opaque zone formation occurs provided us with an estimate to use for birthdate relative to assigning ages and calculating birth month-year and month-year of formation for otolith material.

### Sample collection and otolith processing

Fish samples were collected from U.S. Caribbean waters of PR, STT, and STX during multiple investigations on reef fishes (Fig 2, S1 Table). All fish were measured for fork length (FL mm), total length (TL mm) and weighed (g). Sagittal otolith pairs from each fish were removed, washed in a solution of mild detergent, rinsed, then stored dry in paper coin envelops. To obtain initial age estimates for each fish sample, one otolith from each pair was embedded in resin and then at least two transverse sections (0.5 mm thick) were cut from the embedded otolith through the core using a low speed saw with a diamond edged blade. Sections were glued to glass slides and viewed under a stereoscope at a magnification of 20-60x by two independent readers (S1 Fig). Each reader recorded the number of opaque zones visible on an otolith (one pair of alternating light and opaque zones equals one increment). Only otoliths in which the two readers independently obtained the same increment counts were used for this study.

**Fig 3:**
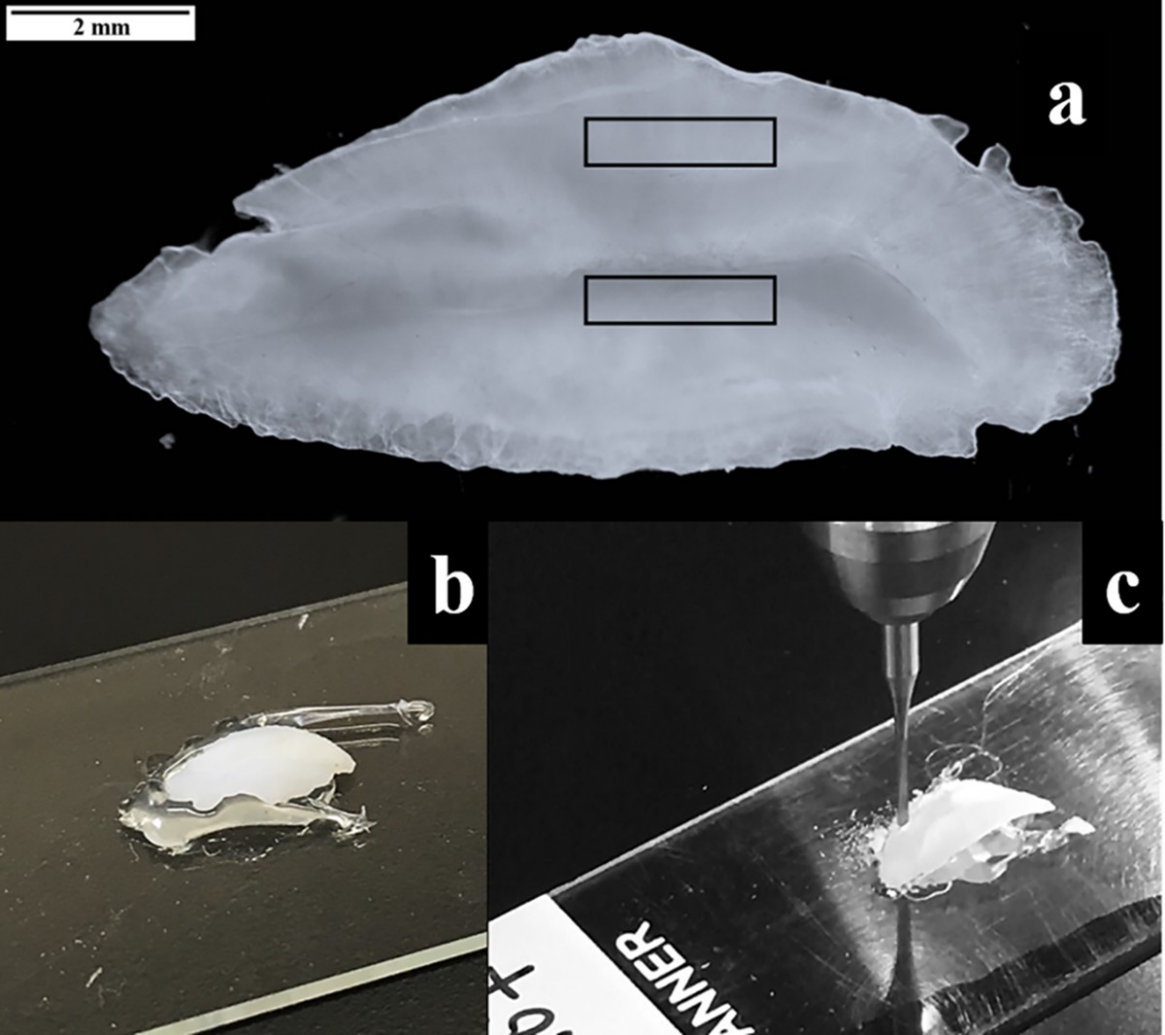
Illustration of steps used to obtain known-age otolith material. The right sagittal otolith of a red hind (a) with rectangles above and below the groove indicating where otolith edge material was removed. Sagittal otoliths were fixed to slides using hot glue (b) and then edge material was milled from the otoliths with a computer-operated micromill (c).

Whole red hind otoliths used for obtaining known-age edge sample material were cleaned in a class 10 clean hood the day prior to processing. Each otolith was flooded with 1% ultrapure HNO3 for 30 s to oxidize any material adhering to their surface, flooded repeatedly with 18.3 MΩ‧cm^-1^ double deionized water to remove acid, and air dried for 12–18 h. Each otolith was then affixed proximal side up with hot glue to a sterilized glass slide (Fig 3). Hot glue was used due to the convex shape of red hind sagittae; a dollop of glue was placed in the middle of the slide, then the otolith was centered on top of the glue which provided a supportive surface along the length of the distal side of the otolith. This prevented major cracking and breaking from the pressure placed on the proximal side of the otolith during the milling process. Otolith edge material was then extracted utilizing a computer automated New Wave Research (ESI-NWR Division; Fremont, CA, USA) micromilling instrument. A 0.3-mm diameter bit was used to mill otolith material along the dorsal and ventral ridges of the sulcal groove using overlapping surface line scans (~2 mm long) conforming to the uneven surface structure of each whole otolith (Fig 3). The drill removed the most recently formed otolith edge material to a depth of approximately 0.2 mm. Once extracted, otolith edge material was stored dry in acid-leached glass vials.

**Fig 4:**
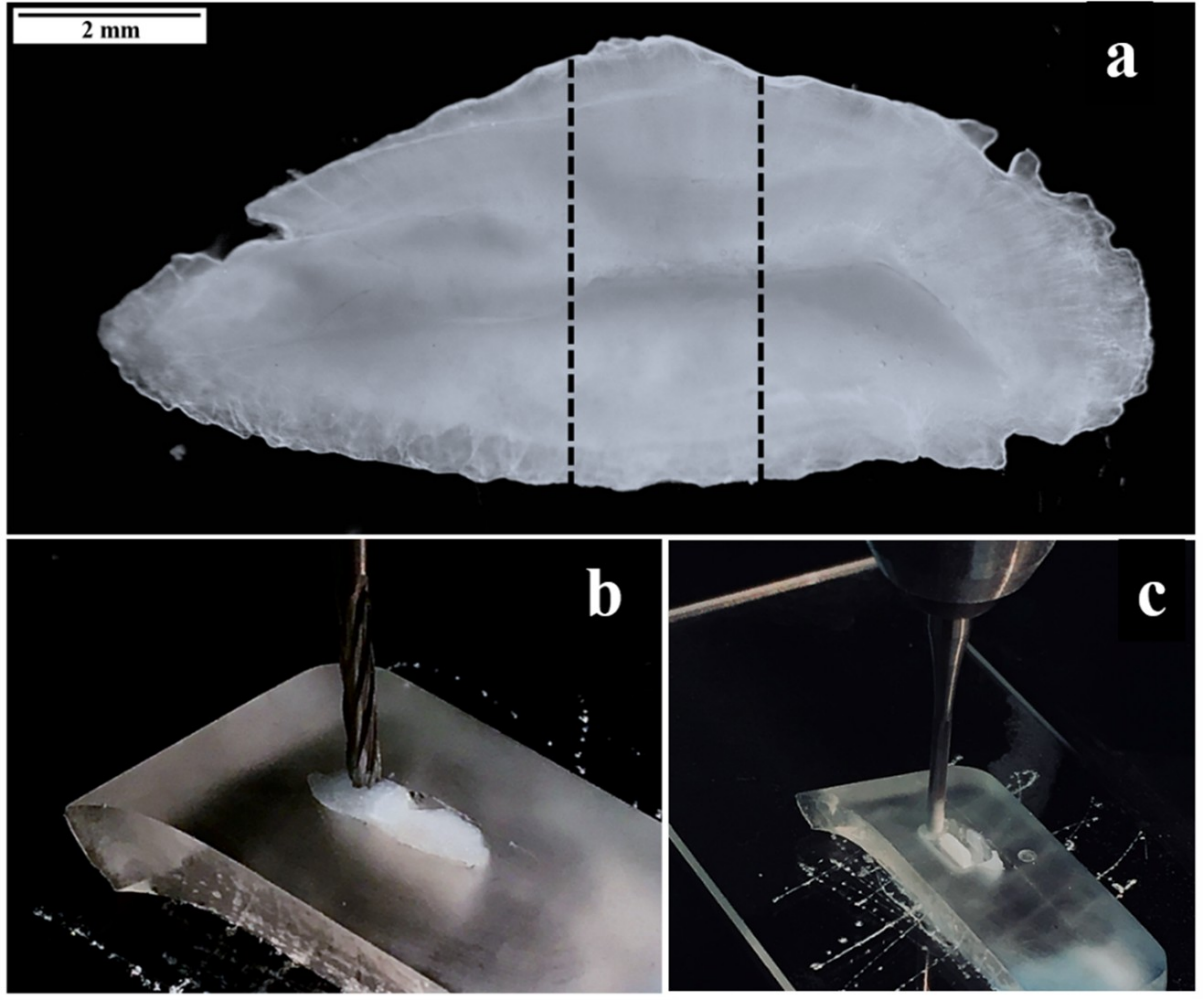
Images illustrating process to obtain red hind otolith core material. The dashed lines on the whole otolith (a) represent the section of the otolith used to extract the core. A core section (b) was glued to a glass slide then the area surrounding the targeted core was first milled by creating a channel surrounding the core material (c).

Otoliths used for core material were cleaned as described above. Once dry, an otolith was embedded in epoxy resin. After the resin had fully hardened, the core section of the embedded otolith was removed by cutting a transverse section at a width of 1.5–2.0 mm that contained the core, then affixed to a glass slide with Loctite glue (Fig 4). The targeted core area included the region from the otolith primordium to the outer edge of the first opaque zone. Using the micromilling system, a channel surrounding the otolith core was drilled, removing all adjacent material from the core area and then the debris generated from this was removed from the section using compressed air. The channel served two purposes. First, the core region of the otolith section was narrow, so creating a channel removed the adjacent material surrounding the core and isolated just the core material for extraction. Second, once the channel was cleaned, it provided a mechanism for trapping the core powder generated from the micromilling process (Fig 4). The core material was then removed by drilling an overlapping surface line scan across its length. Once extracted, core material was weighed to the nearest 0.1 mg and stored dry in acid-leached glass vials.

### AMS analysis

Otolith samples were analyzed for Δ^14^C with accelerator mass spectrometry (AMS) at the National Ocean Sciences Accelerator Mass Spectrometry facility at Woods Hole Oceanographic Institute. Samples were subjected to H_3_PO_4_ in closed, evacuated glass vessels, then produced CO_2_ is stripped and cryogenically purified from H_2_O vapor and N_2_. A portion of the purified CO_2_ is analyzed for ^13^C with isotope ratio-mass spectrometry, and the remainder transferred to a reaction tube and reduced to pure C (graphite) in the presence of H_2_ gas and Fe as a catalyst. The graphite is then analyzed for ^14^C with AMS (additional information on exact methods used can be found online: www.whoi.edu/nosams/radiocarbon-data-calculations). The isotope ^13^C is reported as the delta value δ^13^C (^o^/_oo_), which is calculated as the ratio of ^13^C/^12^C relative to a standard (Pee Dee Belemnite). Radiocarbon (^14^C) is also reported as a delta value (Δ^14^C) that represents the activity of a sample relative to a standard [16] and corrected for age and δ^13^C. Values of Δ^14^C are reported with +/- 1 SD, which incorporates statistical and analytical sources of error.

### Data analyses

A linear regression was computed to test whether a significant relationship existed between red hind known-age otolith Δ^14^C material (edges and whole otolith) and estimated year of material formation. For this analysis, we determined the year of formation value of the red hind otolith edge material by calculating the midpoint in time that the edge material formed based on the date the fish sample was collected and the volume of material extracted from the outer translucent and opaque zones of the otolith. Similar to Kastelle et al. [30], this adjustment assumes constant linear otolith mass accretion during the time the edge material formed. The one red hind whole otolith sample analyzed as part of the known-age samples was from an age-0 fish collected in September 1988. We assumed the fish was spawned during the peak spawning month for red hind (February), which resulted in an estimated midpoint of June 1988.

To determine if known-age otolith material from additional shallow water reef fish species collected from the same region yielded similar Δ^14^C signals, whole otoliths for juvenile Caribbean yellowtail snapper *Ocyurus chrysurus* and white grunt *Haemulon plumierii* were analyzed for Δ^14^C as described above. The linear relationship of the known-age red hind otolith Δ^14^C material was plotted with 95% prediction intervals so that year of formation and Δ^14^C data from the whole otoliths of white grunt and yellowtail snapper juveniles could be superimposed and compared.

The decline period of four reference ^14^C chronologies were used for comparison in this study (Fig 1): one from the east coast of PR [21], one from the southwest coast of PR [22], one from Florida Keys [10, 23], and one from GOM [20]. The two coral-based chronologies from PR consisted of Δ^14^C from annual bands of *O*. *annularis* and overlapped with samples from the current study for the years of 1987–2004. The overlap period for Δ^14^C reference chronology using annual bands of *O*. *annularis* from south Florida was 1987–2010 and extended to 2014 with the inclusion of red hind otolith edge samples collected from the same region and analyzed as part of the current study (S1 Table). The fourth reference chronology was developed for the northern Gulf of Mexico using Δ^14^C of otoliths from known-age red snapper *L*. *campechanus* [20] and overlapped with the current study for the years of 1989–2015. We compared the Δ^14^C linear decline established from our known-age red hind samples with each of the four reference Δ^14^C decline series using pairwise equal slope tests [31] to determine if the decline relationships differed significantly.

The second objective of our study was to use the new region-specific north Caribbean bomb radiocarbon chronometer to validate age estimates of red hind by analyzing Δ^14^C in otolith cores with birth years from the past three decades. This was done through examining potential ageing bias by purposely shifting the estimated ages by +/- 1 to 3 years and superimposing Δ^14^C otolith core values on our known-age red hind otolith reference Δ^14^C time series [20, 32]. The original age estimates represented an age bias of 0 (null model), age biases of +1, +2, +3 shifted age estimates older, and age biases of -1, -2, -3 shifted age estimates to the younger. We then computed the sum of squared residuals (SSR) from predicted versus observed birth years for the cored otolith samples and repeated this for the purposely biased age estimate models [32]. Red hind otolith core material milled for Δ^14^C consisted of the otolith nucleus out to the edge of the first opaque zone. Peak spawning for red hind occurs in February and the formation of the first opaque zone is completed by July of the next year, encompassing 18 months of growth [29]. Estimated birth year equals the year of collection minus the opaque zone count. We adjusted the year of formation value (estimated birth year) for red hind otolith cores by adding 0.83 months to the original birth year estimate (midpoint in time that core material was formed).

The third objective of our study was to test the applicability of the red hind Δ^14^C chronometer for validating age estimation for other bony fishes from the north Caribbean. This was accomplished using otoliths from white grunt and mutton snapper *Lutjanus analis* samples collected in 1988–2020 from across the same sampling region. White grunt and mutton snapper otoliths were processed as described above for age estimation and obtaining otolith core material. The potential for ageing bias was then evaluated as described above. The core material extracted from white grunt and mutton snapper otoliths encompassed the otolith nucleus out to the end of the first translucent zone (prior to the beginning of the first opaque zone). U.S. Caribbean white grunt and mutton snapper peak spawning occurs in June and formation of the first opaque zone starts around June-July. We assumed that the material contained within the milled core formed from beginning of July of the birth year through June of the next year which yielded a midpoint in time of January (six months after peak spawning). Therefore, we adjusted the year of formation value for white grunt and mutton snapper otolith cores by adding + 1 to the original estimate for the birth year. For example, a mutton snapper caught in 2020 with an otolith opaque zone count of 10 would have a year of formation estimate of: 2020[collection year]– 10[otolith opaque zone count] = 2010 + 0.5[fish spawned during peak spawning period of June/July which is the month-based fraction of that estimated birth year] + 0.5[adjustment accounting for midpoint of 1-year time period contained in the otolith core sample] = 2011.

## Results

**Fig 5:**
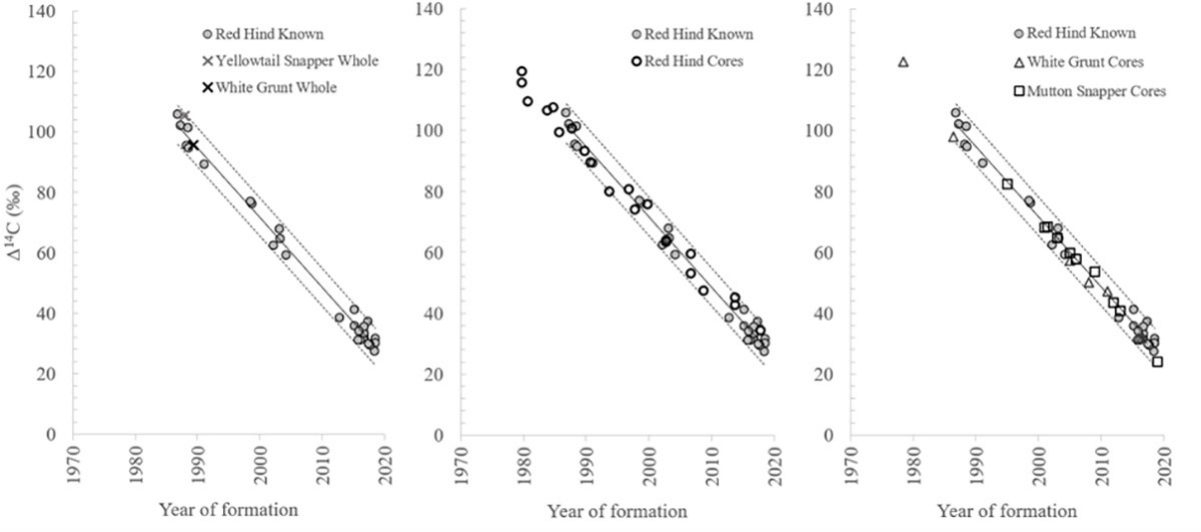
Radiocarbon temporal relationship. Results from known-age red hind otolith material were plotted with results from linear regression analysis (solid line = linear regression fit and dash lines = 95% prediction interval). Graph on far left illustrates whole otoliths of juvenile white grunt and yellowtail snapper sampled in the US Caribbean were analyzed for Δ^14^C and data were overlaid on the known-age red hind Δ^14^C regression results to determine if otoliths from additional species fell within the 95% confidence intervals. Middle graph illustrates results from adult red hind otolith core Δ^14^C versus estimated birth year derived from otolith opaque zone counts for red hind. Graph on right shows Δ^14^C results for otolith cores from adult white grunt, and mutton snapper samples collected in the US Caribbean versus the corresponding opaque zone count-based estimates of fish sample birth years overlaid on the reference series.

Bomb radiocarbon (Δ^14^C) results were obtained from red hind otolith edges for a total of 30 samples collected over the years of 1988–2019 (S1 Table); 10 were sampled from PR (27.3–105.7‰; 1988–2019); nine were from STT (30.1–76.2‰; 1999–2019); seven from STX (29.2–102.0‰; 1998–2019), and four from south Florida (46.3–63.7; 2006–2014). The decline relationship between year of formation for U.S. Caribbean red hind otolith material and Δ^14^C was significant (linear regression: R^2^ = 0.99, p < 0.001; Fig 5, Table 1). The whole otoliths from Caribbean white grunt and yellowtail snapper caught 1988–1989 had Δ^14^C values that fell within the 95% prediction intervals (PI) for red hind (Fig 5).

**Table 1:** Results from statistical analyses.

Analysis	Southwest PR Coral	East PR Coral	South FL Coral	South FL Coral + Otoliths	All GOM Red Snapper	GOM Red Snapper	Known-Age Red Hind
Time Period	1977–2004	1979–2004	1977–2010	1977–2014	1989–2016	1989–2016	1987–2019
Linear Relationship	y = 4799–2.36x	y = 5144–2.53x	y = 5383–2.65x	y = 5360–2.64x	y = 5206–2.56x	y = 5230–2.58x	y = 4680–2.30x
R^2^; *P*	0.93; < 0.001	0.98; < 0.001	0.98; < 0.001	0.98; < 0.001	0.96; < 0.001	0.98; < 0.001	0.99; < 0.001
	n = 23	n = 41	n = 35	n = 39	n = 21	n = 20	n = 27
Difference	23.4–24.7‰	13.3–13.8‰	16.3–17.2‰	16.2–17.0‰	18.9–20.4‰	14.9–16.2‰	12.4–12.9‰
95% PI							
Equal Slopes Test							
Overlap Years	1987–2004	1987–2004	1987–2005	1987–2014	1989–2016	-	-
Test Statistic; *P*	t = -2.4; 0.02	t = -62.5; < 0.001	t = -6.0; < 0.001	t = -6.1; < 0.001	t = -7.2; < 0.001		
	n = 18	n = 30	n = 17	n = 25	n = 21		

The first row reports the results from linear regression analysis for each reference set: “Time Period” is the years that were included in the regression; “Linear Relationship” refers to the regression equation; and the sample size for the regression (n), R^2^ and *P* are provided for each regression analysis. The second row reports the difference between the upper and lower values of the individual Δ^14^C prediction intervals (PI). The third row provides the results for the pairwise equal slopes test between the known-age red hind Δ^14^C decline and each reference Δ^14^C decline dataset: the “Overlap Years” indicate the range of years that the two datasets overlap; “Test Statistic” result is provided for each comparison; and the number of samples available for each reference dataset (n) for the overlapping time period with the known-age samples from the current study. Regression results are reported for two versions of the GOM red snapper data: “All GOM Red Snapper” includes the complete temporal series reported in Barnett et al. [20] for the overlapping time period; and “GOM Red Snapper” excludes one data point that was identified as an extreme outlier and could have been a contaminated sample.

**Fig 6:**
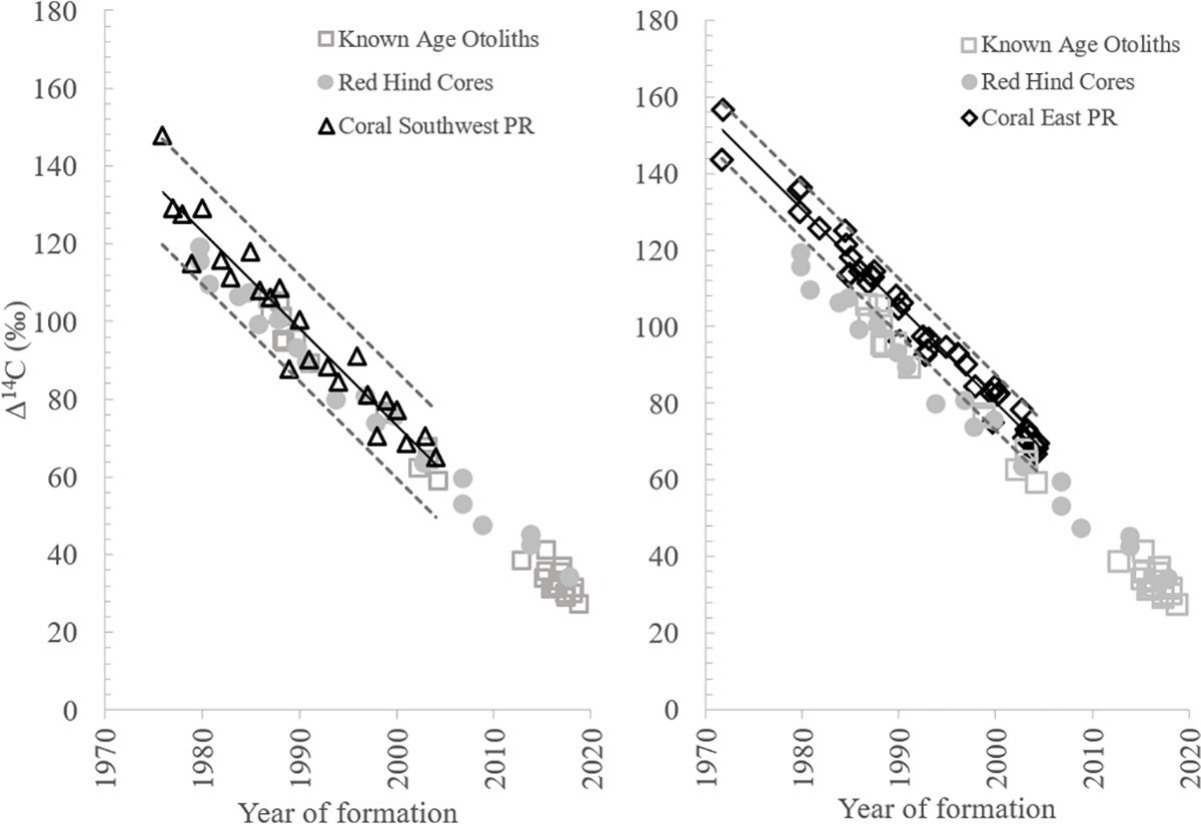
Puerto Rico coral-based bomb radiocarbon results during decline period. Coral-based bomb radiocarbon decline relationships for the two Puerto Rico reference studies overlaid on data from the current study for the red hind known-age otoliths and otolith cores. Dashed lines represented the 95% prediction intervals for the linear regression analysis for each reference dataset. Corresponding linear regression analysis results are detailed in Table 1.

**Fig 7:**
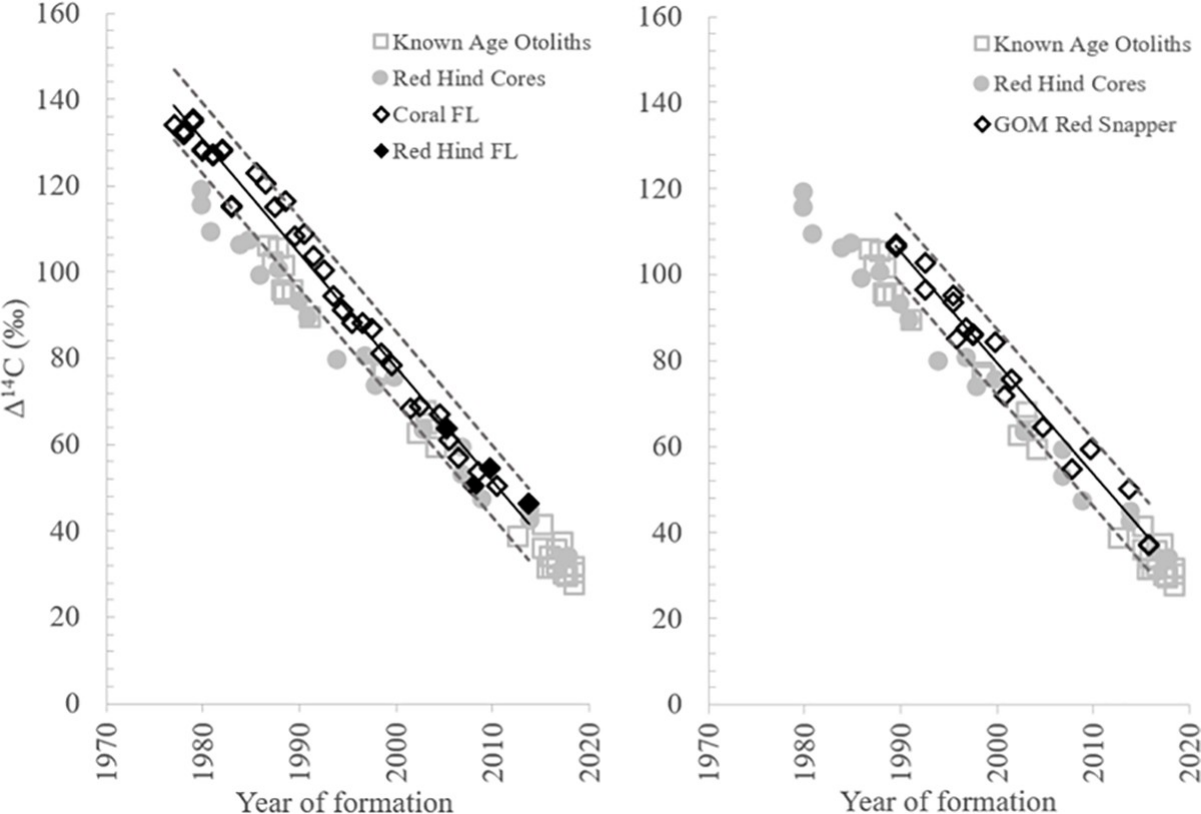
Bomb radiocarbon decline relationships from south Florida and GOM. Bomb radiocarbon decline relationships from the south Florida coral series and the nGOM red snapper known-age otolith series overlaid on data from the current study for red hind known-age otolith and otoliths cores. Dashed lines represented the 95% prediction intervals for the linear regression analysis for each reference dataset. Corresponding linear regression analysis results are detailed in Table 1.

The known-age Caribbean red hind Δ^14^C decline relationship differed significantly from the Δ^14^C decline relationships for the four reference datasets (Figs 6 and 7, Table 1). The Δ^14^C values for red hind edges and whole otolith material fell within the 95% PI for Δ^14^C of the southwest PR coral samples during the overlapping time period of the two studies (Table 1; Fig 6), but the red hind Δ^14^C decline slope was slightly, but significantly different (P = 0.02; Table 1: red hind slope = -2.30 and southwest PR slope = -2.36). The difference between the Δ^14^C PI values for southwest PR coral was greater than the red hind PI (~24‰ versus ~13‰; Table 1), indicating more variability around the regression line occurred for the coral results.

The slope of the Δ^14^C red hind decline was significantly different from the east PR Δ^14^C coral decline relationship with the majority of Δ^14^C red hind samples exhibiting depleted values relative to east PR coral samples during the overlapping time period (Table 1, Fig 6). The red hind slope also differed significantly from the south FL Δ^14^C decline and the GOM red snapper Δ^14^C decline during the overlapping time periods (Table 1), and red hind Δ^14^C values were relatively depleted compared to the south FL samples and GOM red snapper (Fig 7). From these results combined, the known-age Caribbean red hind Δ^14^C decline relationship provides a more comprehensive decline series for the north Caribbean and extends the Δ^14^C time series in that region from 2004 to 2019. Additionally, the prediction interval differences for east PR and south Florida coral Δ^14^C decline series were similar to red hind Δ^14^C decline (~13‰ for east PR and ~16‰ for south Florida; Table 1).

Red hind age estimates ranged from 1 y for a sample collected from PR in 2019 (163 mm TL) to 17 y for a sample from STT collected in 2016 (354 mm TL; S1 Table). White grunt age estimates ranged from 2 y for a sample caught from STX in 1988 (190 mm FL) to 14 y for two 2019 samples from STT (324 mm and 353 mm FL). Mutton snapper age estimates ranged from 1 y for a sample caught from PR in 2019 (290 mm FL) to 19 y for a sample caught in 2019 from STT (624 mm FL). Results from the ageing bias analysis of red hind otolith cores Δ^14^C values relative to the regression fit of the known-age red hind Δ^14^C decline indicated red hind birth year estimates derived from sagittal otolith thin section opaque zone counts are accurate, given that the original age estimates had the lowest SSR (217), while the purposefully biased age estimates resulted in SSR values ranging from 231 for -1 y to 1254 for +3 y (Table 2). Caribbean white grunt otolith opaque zone counts resulted in corresponding birth years that fell within the 95% PI of the known-age red hind Δ^14^C decline (Fig 5). Ageing bias analysis results for white grunt indicated that the original, unadjusted age estimates were accurate because they had the lowest SSR at 50 (Table 2). Similarly, for mutton snapper otolith opaque zone counts resulted in corresponding birth years that fell within the 95% PI of the known-age red hind Δ^14^C decline (Fig 5). Ageing bias analysis results for mutton snapper indicated that the original, unadjusted age estimates were accurate because they had the lowest SSR at 32 (Table 2).

**Table 2:** Results from ageing bias analysis.

Age Model	Bias applied years	Red Hind SSR	White Grunt SSR	Mutton Snapper SSR
Null	0	217	50	32
-1	-1	383	109	130
-2	-2	729	273	333
-3	-3	1254	542	643
+3	+3	799	511	374
+2	+2	425	251	155
+1	+1	231	98	41

Birth year estimates were purposefully biased by +/- 1 to 3 years for each species and then the squared residuals from the predicted known-age red hind otolith reference Δ^14^C time series regression were computed.

## Discussion

Red hind known-age otoliths from the north Caribbean region provided a significant linear decline record of DIC Δ^14^C for shallow waters (< 100 m depth). This regional reef fish otolith Δ^14^C chronometer extends the previous Δ^14^C record from the north Caribbean for an additional 15 y (from 2004 to 2019), past the change-over period (post 2000) of the bomb era to the fossil fuel era [23]. Our results provide additional Δ^14^C data that could be used for investigations on oceanic water mass age and renewal and a new Δ^14^C reference chronometer that can be used as an important tool to validate age estimation for a multitude of reef fishes from the north Caribbean region. The current study is the first to use Δ^14^C to validate age estimates of tropical Atlantic fisheries species populations from the Caribbean and the first study to use Δ^14^C to validate ageing methods specifically for red hind, white grunt, and mutton snapper. All three of these are economically important fisheries species in the Caribbean.

The red hind Δ^14^C temporal decline differed significantly from the reference coral Δ^14^C declines reported for southwest PR [22], northeast PR [21], and south FL [10, 23] and from the Δ^14^C temporal relationship reported for GOM red snapper otoliths [20]. Selection of a Δ^14^C reference chronology is an important initial step in assessing fish ageing error. Early on in the history of research utilizing bomb radiocarbon for fish ageing validation, Δ^14^C reference chronologies were limited, but in the past 20 years additional datasets have been developed for multiple regions, so fisheries investigators are no longer extremely limited to a few reference chronologies. The sources of Δ^14^C reference data for ageing validation work are varied, and consist of Δ^14^C measured directly from the atmosphere [33], from known-age otoliths [20], from otolith core Δ^14^C with validated ages [30, 34, 35], and from Δ^14^C reported from corals [19, 20]. To explain our findings within the context of past research involving the use of temporal Δ^14^C trends and to emphasize the applicability of the north Caribbean reference chronology we established in this study, we identified three main considerations that future ageing validation studies should evaluate to strengthen their findings: 1) evaluation of the applicability of the original goal/objectives and study design of potential Δ^14^C reference studies; 2) evaluation of the applicability of potential Δ^14^C reference data to the region/location where fish samples under evaluation were obtained; and 3) determination of the location of habitat in which the fish species under investigation spends at least the juvenile period that was recorded in the otolith core material extracted for Δ^14^C analysis. These considerations expand on some past assumptions identified when using the Δ^14^C fish ageing validation method [30, 33, 36–38], while also incorporating insights from our current study.

A reference Δ^14^C chronology for the purpose of ageing validation work provides a temporal series of the expected annual ranges of Δ^14^C levels for the waters a fish experienced during the time its otolith core formed. A reference Δ^14^C chronology with narrow 95% PIs during the decline period would prove particularly useful when validating fish age estimates for more recently collected samples with estimated birth years after 1975 or for fish species with relatively short lifespans (< 20 y). Many potential coral-based radiocarbon chronologies originate from research focused on documenting uptake of industrial CO_2_ [10], determining water mass age, turnover time, tracing contributions of source waters, or quantifying growth rates and ages of coral taxa by analyzing annual depositions of carbonate material in corals [9, 21, 22, 39–41]. Therefore, the original objectives and study designs of these various types of investigations will dictate the usefulness and applicability of resulting Δ^14^C data for the purpose of fish ageing validation.

Two investigations from north Caribbean waters reported coral-based Δ^14^C temporal series that differed distinctly from each other and both studies utilized single coral cores from one coral head within their study sites [21, 22]. Our known-age red hind otolith Δ^14^C chronology differed significantly from these two PR coral-based Δ^14^C series. The differences can be partially explained by evaluating the applicability of the objectives and methods for the coral studies; the research goals for the two coral-based studies were disparate from each other, as were the sampling targets for obtaining coral material for Δ^14^C analysis. Originally, we selected the east PR Δ^14^C time series for comparison with fish otoliths results because it was used previously in GOM fish ageing validation work as part of a larger Δ^14^C chronometer compiled from several coral-based studies from a large area in the western Atlantic. However, the focus of the east PR investigation was to document the terrestrial input of carbon delivered by a tropical riverine system to adjacent nearshore, coastal waters [21]. The one coral core obtained to measure Δ^14^C levels through time was selected because of its proximity to freshwater input, and coral material for Δ^14^C analysis was only obtained for coral bands representing years in which large negative δ^13^C excursions had been measured across the overall growth period of 1948–2004 represented in the coral core [21]. The selective measurement of Δ^14^C exclusively from coral bands for only years of depleted δ^13^C did not produce a Δ^14^C chronology that represents overall DIC Δ^14^C trends from shallow marine waters [23], thus the east PR study does not seem appropriate in the context of ageing validation for reef fish species from the north Caribbean.

The research objective of the southwest PR study was to examine decadal- and interannual-scale variations in the contributions from tropical and subtropical source waters to the study site; coral material for Δ^14^C analysis was obtained from each annual coral band for the years of 1950–2004 [22]. The Δ^14^C from the one coral core sampled for the study had a peak Δ^14^C of 147.7‰ in 1976; the southwest PR coral Δ^14^C decline period documented a wide range of variability at the start of the decline period (Fig 6). Although the red hind Δ^14^C otolith values were all within the 95% PI of the southwest PR coral Δ^14^C relationship, our known-age otolith Δ^14^C decline differed in two important ways: 1) the decline slope was slightly but significantly different between studies; and 2) annual variability of Δ^14^C for the coral relationship resulted in a large difference for year of formation and Δ^14^C 95% prediction intervals (~24‰) compared to the red hind Δ^14^C decline (~13‰; Table 1).

The southwest PR coral Δ^14^C chronology provided useful results for the objective of the original study on decadal-scale source water variation [22], but the coral Δ^14^C results would not have been as useful in fish ageing validation work. Some of the differences between coral and otolith Δ^14^C values for specific years may relate to the different methods used to extract CaCO_3_ material in each study. Kilbourne et al. [22] used a Dremel tool with 1-mm bit mounted on a computer-controlled drilling stage. The current study used a New Wave Research micromilling instrument with a 0.3 mm diameter bit to precisely mill otolith edge material. The Dremel system may be less precise in obtaining just the targeted material from coral bands, compared to the micromilling instrument which enable us to control the depth of penetration at a scale of 0.05 mm for each pass of the drill. Another potential explanation as to why the known-age otolith Δ^14^C decline differed from the southwest PR coral record is because the fish samples originated from multiple locations throughout the northern Caribbean in the well-mixed shallow waters of red hind coral reef habitat, while the one coral core sample came from a single site, located ~3 km from shore, off of southwestern corner of PR (Fig 1) and reflected the environmental and oceanographic factors (and associated variability) experienced at that site, which was selected in part because of its proximity to potential sources of upwelling [22]. Caribbean oceanic inflow comes from a combination of north Atlantic and south Atlantic origins [42–44]. The contributions to north Caribbean waters from these sources vary spatially and temporally [22, 45]. The coral head from which the one core was extracted in that single location recorded the site-specific seawater Δ^14^C levels through time [22]. The red hind otolith edge material would have recorded Δ^14^C experienced by the fish as it moved around within the shallow waters of the coral reef system contained within its range.

Another important consideration that we identified through the course of our current investigation, and is relevant to future application of our north Caribbean reference decline series, is the applicability of potential Δ^14^C reference data to the region/location where fish samples under evaluation were obtained [38]. The known-age otolith Δ^14^C results from the current study combined with the southwest PR coral Δ^14^C reported in Kilbourne et al. [22] demonstrated that a regional difference exists between the north Caribbean and south Florida Δ^14^C chronologies. Several other investigations also have documented regional differences in temporal trends of Δ^14^C reported from CaCO_3_ material (summarized in Toggweiler et al. [23]). Druffel [39] investigated Δ^14^C levels over time from the annual bands of two coral species from Bermuda, *Diploria strigosa* and *D*. *labryinthiformis* and compared the results to Δ^14^C obtained from annual bands of *O*. *annularis* collected from south Florida [10, 46]. Druffel [39] noted “striking dissimilarities” between the post-1950 Δ^14^C chronologies between Bermuda and Florida corals and attributed those differences to site-specific mixing processes. Kastelle et al. [30] evaluated differences in basin Δ^14^C reference otolith chronologies from the northeastern Pacific and demonstrated that using a Δ^14^C reference chronology derived from a different ocean basin to validate age estimation of a fish population could result in erroneous interpretation of ageing bias.

An extensive review of the global distributions of Δ^14^C from oceanic sources identified regional differences due to upwelling patterns in oceanic basins north of the Antarctic Circumpolar Current [23]. Average Δ^14^C was calculated for multiple “regions of interest” over three time periods: 1940–1954, 1990–1994, and 2003–2005. The regional averages for each time period were then normalized by subtracting the contemporary Δ^14^C value of Okinawa in the north Pacific resulting in region/time period-specific “deficit” values. Toggweiler et al. [23] reported that for regions of the western Atlantic, in waters of North America and the Caribbean, major differences occurred in Δ^14^C deficits during the three time periods. For example, during the time period of 1990–1994, the deficits for Bermuda, Florida, and Caribbean were -3, -20, and -25 respectively. Toggweiler et al. [23] utilized southwest PR Δ^14^C data [22] to calculate the deficits for the Caribbean region. A similar difference of 5‰ in mean Δ^14^C exists between north Caribbean known-age otoliths from the current study and south Florida corals for 1990–1999; north Caribbean average Δ^14^C was 87‰; south Florida average Δ^14^C was 92‰ [23].

Past research on age and growth in fish species that utilized Δ^14^C reference data from outside the region of investigation may benefit from re-examining the Δ^14^C results for their samples. For example, a study on life history of barrelfish *Hyperoglyphe perciformis* from the southeastern U.S., 140 km southeast of Charleston, SC [47], included ageing validation of the species using Δ^14^C reference series from the northeastern Atlantic near Newfoundland [48] and Δ^14^C results for red snapper otolith cores obtained to validate ageing for red snapper from the northern Gulf of Mexico [49]. The northeastern Atlantic region off Newfoundland had a Δ^14^C deficit of -26 during the pre-bomb (1940–1954) period, compared to -7 for Bermuda and -17 for Florida, two regions in closer proximity to the Charleston Bump [23]. The regional difference in Δ^14^C for Newfoundland waters was even more pronounced from 1990–1994 at -65 compared to Bermuda (-3) and Florida (-20; [23]).

Researchers have emphasized when using the Δ^14^C method to validate ageing, a basic and important assumption is that the reference chronology must be biologically and environmentally representative of the species under evaluation [33, 36, 38]. The third consideration that we identified expands on this assumption in emphasizing that the timing and magnitude of the reference Δ^14^C temporal relationship should reflect the Δ^14^C experienced by a fish in the habitat where it resided as a juvenile, when the otolith core material formed. Several marine fisheries species are known to exhibit ontogenetic shifts in habitat, utilizing shallower, inshore areas within a region as juveniles then moving to deeper habitat further offshore as adults [50–53]. Therefore, understanding the ontogenetic shifts in habitat use for a species is important when considering the application of the Δ^14^C chronometer for ageing validation.

Our study applied the north Caribbean reference Δ^14^C chronology derived from known age otoliths to validate ageing for red hind, white grunt, and mutton snapper, three species known to occur as juveniles in relatively shallow areas within or adjacent to the coral reef habitat where adults occur [54–56]. The juvenile habitats for our three species in the north Caribbean are characterized by the same water chemistry of the reef system as a whole. However, some areas within a regional seascape have known (and unknown) inputs of depleted Δ^14^C; known gradients of Δ^14^C occur in association with freshwater input [57, 58], upwelling [23, 59], and depth [60–62]. Andrews et al. [57] observed anomalies in the Δ^14^C otolith core levels of gray snapper *Lutjanus griseus* collected from the eastern GOM and determined that the estuarine waters of west Florida gray snapper juvenile habitat were Δ^14^C-depleted relative to the marine waters where adults were collected. Waters of the nearshore, estuarine habitat of juvenile gray snapper contained a mixture of sources including riverine freshwater, Δ^14^C-depleted groundwater, and marine water which resulted in an overall depleted Δ^14^C signature [57].

The location of juvenile habitat within a regional seascape remains unknown or undocumented for many fisheries species. Applying our north Caribbean Δ^14^C chronometer to validate ageing using otolith cores for fish species with unknown juvenile habitat could result in the misinterpretation of Δ^14^C results and inconclusive results on potential ageing error. A few studies on fish species typically caught in deeper shelf and slope habitats (depths far exceeding 100 m), but for which juvenile habitat remains unknown, reported Δ^14^C results that deviated from the expected age estimate-derived birth years [47, 63, 64]. These studies concluded that Δ^14^C results validated ageing methods for the species and that the depleted Δ^14^C values from otolith cores may indicate that the juveniles for each species utilized deeper, Δ^14^C-depleted habitat or habitat impacted by upwelling of deeper Δ^14^C water. However, these conclusions seem speculatory and until the juvenile habitat is documented for these species, the conclusion that ageing methods were validated is premature.

Future investigations involving Δ^14^C for ageing validation of deepwater species from the north Caribbean could alternatively utilizes eye lens cores to obtain birth year Δ^14^C instead of using otolith cores [65–67]. Fish eye lenses are similar to otoliths in that they continue to grow throughout the life of a fish, once formed are metabolically inert [67], and contain isotopic histories of ontogenetic shifts in habitat [68, 69]. In addition, eye lens cores are derived from 100% metabolic carbon sources [65]. Organic carbon Δ^14^C of deep oceanic organisms is mainly derived from phytoplankton in the euphotic zone [61, 62] so eye lens cores would reflect a similar Δ^14^C as the reference chronologies established from shallow water species.

## Conclusions

Overall, results from this study have broad implications for fishery management in the Caribbean. Accurate age estimation is fundamental to estimating population parameters for fisheries species and also important for estimating sustainable harvest rates. Through our research, we used known-age otolith material from red hind to establish a region-specific north Caribbean Δ^14^C reference chronometer, demonstrated that it was different from two coral-based Δ^14^C temporal series reported from PR, from the coral-based Δ^14^C chronology from the adjacent region of south Florida, and from GOM known-age red snapper Δ^14^C temporal results from the decline period. Our north Caribbean Δ^14^C reference chronometer was successfully applied to validate ageing methods for three economically important reef fish species; results indicated opaque zone counts on transverse otolith thin sections provide accurate age estimates for red hind, white grunt, and mutton snapper from Caribbean waters. During this investigation, we developed three important considerations to strengthen the findings of future age validation studies: 1) the applicability of the original goal/objectives and study design of potential Δ^14^C reference studies; 2) the applicability of potential Δ^14^C reference data to the region/location where fish samples under evaluation were obtained; and 3) the location of habitat in which the fish species under investigation spends at least the juvenile period that was recorded in the otolith core material extracted for Δ^14^C analysis. The second and third considerations are particularly important for ageing validation work utilizing our north Caribbean Δ^14^C reference chronometer.

## Supporting information

S1 Table Otolith samples from Caribbean fishes analyzed for Δ^14^C with AMS. Sample type includes adult core, edge sample of adult otolith, or whole juvenile otoliths. Otoliths for which no fish size was recorded are shown as -. Reported length for red hind was total length (TL). Reported length for all other species was fork length (FL).
(DOCX)

S1 Fig Otolith sections of red hind samples viewed with reflected light. The top otolith section is from a 3 year old red hind and the bottom otolith section is from a 17 year old red hind.
(TIF)
