# Supplementary material for: Radiocarbon in otoliths of tropical marine fishes: Reference Δ14C chronology for north Caribbean waters
Source: PLoS One. 2021 May 12;16(5):e0251442. doi: 10.1371/journal.pone.0251442 (PMC8115809; doi:10.1371/journal.pone.0251442)
Supplement: S1 Table — Sample type includes adult core, edge sample of adult otolith, or whole juvenile otoliths. Otoliths for which no fish size was recorded are shown as -. Reported length for red hind was total length (TL). Reported length for all other species was fork length (FL). (DOCX) [file pone.0251442.s001.docx]

S1 Table. Otolith samples from Caribbean fishes analyzed for Δ^14^C with AMS. Sample type includes adult core, edge sample of adult otolith, or whole juvenile otoliths. Otoliths for which no fish size was recorded are shown as -. Reported length for red hind was total length (TL). Reported length for all other species was fork length (FL).

| **Species** | **Sample Type** | **Island** | **ID** | **Year of Collection** | **FL/TL (mm)** | **Year of Formation** | **Age (y)** | **δ^13^C ‰** | **Δ^14^C ‰** | **SE** |
| --- | --- | --- | --- | --- | --- | --- | --- | --- | --- | --- |
| red hind | edge | PR | RH-PR-01 | 1988 | 212 | 1986.8 | 3 | -3.23 | 105.7 | 2.2 |
| red hind | edge | PR | RH-PR-02 | 1989 | 225 | 1988.6 | 2 | -3.89 | 94.7 | 2.2 |
| red hind | edge | PR | RH-PR-03 | 1990 | 309 | 1988.3 | 3 | -3.13 | 95.4 | 2.1 |
| red hind | edge | PR | RH-PR-04 | 1992 | 251 | 1991.2 | 2 | -3.44 | 89.3 | 2.1 |
| red hind | edge | PR | RH-PR-05 | 2013 | 230 | 2012.8 | 2 | -4.00 | 38.5 | 2.0 |
| red hind | edge | PR | RH-PR-06 | 2016 | 280 | 2016.2 | 3 | -2.27 | 31.1 | 2.0 |
| red hind | edge | PR | RH-PR-07 | 2016 | 265 | 2015.3 | 4 | -3.79 | 35.7 | 2.0 |
| red hind | edge | PR | RH-PR-08 | 2017 | 193 | 2016.8 | 2 | -5.07 | 35.6 | 2.1 |
| red hind | edge | PR | RH-PR-09 | 2018 | 275 | 2017.5 | 3 | -2.49 | 29.9 | 2.4 |
| red hind | edge | PR | RH-PR-10 | 2019 | 300 | 2018.5 | 3 | -3.22 | 27.3 | 2.0 |
| red hind | edge | STT | RH-STT-01 | 1999 | - | 1998.8 | 3 | -2.74 | 76.9 | 2.1 |
| red hind | edge | STT | RH-STT-02 | 2000 | 360 | 1998.8 | 6 | -1.93 | 76.2 | 2.1 |
| red hind | edge | STT | RH-STT-03 | 2003 | 304 | 2002.3 | 5 | -2.02 | 62.4 | 2.2 |
| red hind | edge | STT | RH-STT-04 | 2004 | 351 | 2003.2 | 5 | -2.39 | 67.7 | 2.1 |
| red hind | edge | STT | RH-STT-05 | 2015 | 291 | 2015.3 | 3 | -3.15 | 41.2 | 2.0 |
| red hind | edge | STT | RH-STT-06 | 2016 | 382 | 2016.0 | 6 | -2.03 | 33.9 | 2.5 |
| red hind | edge | STT | RH-STT-07 | 2017 | 285 | 2016.8 | 4 | -3.72 | 33.3 | 1.9 |
| red hind | edge | STT | RH-STT-08 | 2018 | 238 | 2017.4 | 3 | -3.55 | 37.1 | 2.2 |
| red hind | edge | STT | RH-STT-09 | 2018 | 264 | 2018.6 | 2 | -2.23 | 30.1 | 2.0 |
| red hind | edge | STX | RH-STX-01 | 1988 | 245 | 1987.3 | 4 | -2.55 | 102.0 | 2.3 |
| red hind | edge | STX | RH-STX-02 | 2004 | 279 | 2003.3 | 4 | -1.95 | 64.6 | 2.5 |
| red hind | edge | STX | RH-STX-03 | 2005 | 252 | 2004.3 | 5 | -1.56 | 59.1 | 3.1 |
| red hind | edge | STX | RH-STX-04 | 2016 | 283 | 2015.8 | 4 | -2.48 | 31.1 | 2.1 |
| red hind | edge | STX | RH-STX-05 | 2017 | 275 | 2016.8 | 4 | -2.31 | 32.0 | 3.1 |
| red hind | edge | STX | RH-STX-06 | 2018 | 268 | 2017.8 | 4 | -3.13 | 29.2 | 2.0 |
| red hind | edge | STX | RH-STX-07 | 2019 | 280 | 2018.6 | 3 | -4.88 | 31.5 | 7.5 |
| red hind | edge | FL | RH-FL-01 | 2006 | 380 | 2005.3 | 8 | -3.36 | 63.7 | 2.1 |
| red hind | edge | FL | RH-FL-02 | 2008 | 378 | 2008.1 | 9 | -2.66 | 50.5 | 2.0 |
| red hind | edge | FL | RH-FL-03 | 2010 | 345 | 2009.7 | 6 | -2.21 | 54.5 | 2.2 |
| red hind | edge | FL | RH-FL-04 | 2014 | 430 | 2013.8 | 12 | -2.88 | 46.3 | 2.1 |
| red hind | whole | PR | RH-PR-11 | 1988 | 87 | 1988.5 | 0 | -3.28 | 101.3 | 3.3 |
| red hind | core | PR | RH-PR-12 | 1988 | 387 | 1979.8 | 9 | -3.55 | 119.3 | 2.2 |
| red hind | core | PR | RH-PR-13 | 1988 | 473 | 1980.8 | 8 | -3.95 | 115.7 | 2.4 |
| red hind | core | PR | RH-PR-14 | 1988 | 465 | 1980.8 | 8 | -4.05 | 109.3 | 2.3 |
| red hind | core | PR | RH-PR-15 | 1988 | 253 | 1985.8 | 3 | -4.25 | 99.1 | 2.2 |
| red hind | core | PR | RH-PR-16 | 1989 | 358 | 1983.8 | 6 | -5.49 | 106.6 | 2.2 |
| red hind | core | PR | RH-PR-17 | 2016 | 414 | 2002.8 | 14 | -5.40 | 63.4 | 2.1 |
| red hind | core | PR | RH-PR-18 | 2016 | 335 | 2008.8 | 8 | -4.47 | 47.4 | 2.0 |
| red hind | core | PR | RH-PR-19 | 2016 | 245 | 2013.8 | 3 | -4.12 | 45.1 | 3.1 |
| red hind | core | PR | RH-PR-20 | 2019 | 163 | 2018.8 | 1 | -4.68 | 34.3 | 5.4 |
| red hind | core | PR | RH-PR-21 | 2019 | 292 | 2013.8 | 6 | -4.15 | 42.6 | 2.1 |
| red hind | core | STT | RH-STT-10 | 1999 | - | 1984.8 | 15 | -3.93 | 107.5 | 2.3 |
| red hind | core | STT | RH-STT-11 | 2000 | 425 | 1993.8 | 7 | NR | 79.7 | 2.4 |
| red hind | core | STT | RH-STT-12 | 2003 | 435 | 1987.8 | 16 | -3.96 | 100.8 | 2.4 |
| red hind | core | STT | RH-STT-13 | 2003 | 402 | 1990.8 | 13 | -4.53 | 89.5 | 2.8 |
| red hind | core | STT | RH-STT-14 | 2003 | 340 | 1996.8 | 7 | -4.03 | 80.8 | 3.4 |
| red hind | core | STT | RH-STT-15 | 2004 | 442 | 1989.8 | 15 | -3.93 | 93.3 | 2.6 |
| red hind | core | STT | RH-STT-16 | 2004 | 382 | 1997.8 | 7 | -4.36 | 73.8 | 3.0 |
| red hind | core | STT | RH-STT-17 | 2015 | 411 | 2002.8 | 13 | -4.24 | 63.8 | 2.1 |
| red hind | core | STT | RH-STT-18 | 2016 | 354 | 1999.8 | 17 | -4.42 | 75.3 | 2.9 |
| red hind | core | STX | RH-STX-08 | 2016 | 437 | 2006.8 | 10 | -4.97 | 53.1 | 2.2 |
| red hind | core | STX | RH-STX-09 | 2016 | 377 | 2006.8 | 10 | -3.60 | 59.6 | 3.4 |
| yellowtail snapper | whole | PR | YS-PR | 1988 | 44 | 1988.0 | 0 | -1.39 | 105.2 | 3.4 |
| white grunt | whole | PR | WG-PR-01 | 1989 | 50 | 1989.8 | 0 | -3.37 | 95.5 | 2.5 |
| white grunt | core | STX | WG-STX-01 | 1988 | 190 | 1987.0 | 2 | -2.27 | 98.0 | 2.4 |
| white grunt | core | STX | WG-STX-02 | 1988 | 193 | 1979.0 | 10 | -0.83 | 122.5 | 2.2 |
| white grunt | core | STX | WG-STX-03 | 2018 | 265 | 2009.0 | 10 | -5.23 | 50.2 | 2.3 |
| white grunt | core | STX | WG-STX-04 | 2019 | 277 | 2014.0 | 6 | -2.61 | 36.1 | 2.1 |
| white grunt | core | STT | WG-STT-01 | 2019 | 269 | 2017.0 | 3 | NR | 31.9 | 2.9 |
| white grunt | core | STT | WG-STT-02 | 2019 | 285 | 2017.0 | 3 | -3.91 | 31.8 | 2.2 |
| white grunt | core | STT | WG-STT-03 | 2019 | 285 | 2007.0 | 13 | -2.92 | 57.4 | 2.1 |
| white grunt | core | STT | WG-STT-04 | 2019 | 310 | 2012.0 | 8 | -2.69 | 47.1 | 2.2 |
| white grunt | core | STT | WG-STT-05 | 2019 | 317 | 2006.0 | 14 | -1.84 | 59.5 | 2.1 |
| mutton snapper | core | PR | MS-PR-01 | 2019 | 566 | 2013.0 | 7 | -1.21 | 40.8 | 4.3 |
| mutton snapper | core | PR | MS-PR-02 | 2019 | 606 | 2003.0 | 17 | NR | 64.7 | 2.2 |
| mutton snapper | core | PR | MS-PR-03 | 2019 | 664 | 2006.0 | 14 | -3.11 | 57.8 | 2.1 |
| mutton snapper | core | PR | MS-PR-04 | 2019 | 205 | 2019.0 | 1 | -1.86 | 24.0 | 2.3 |
| mutton snapper | core | STT | MS-STT-01 | 2019 | 624 | 2001.0 | 19 | -4.01 | 68.3 | 2.2 |
| mutton snapper | core | STT | MS-STT-02 | 2016 | 510 | 2012.0 | 5 | -2.51 | 43.4 | 2.3 |
| mutton snapper | core | STT | MS-STT-03 | 2018 | 588 | 2001.0 | 18 | -0.52 | 68.1 | 2.2 |
| mutton snapper | core | STT | MS-STT-04 | 2019 | 565 | 2009.0 | 11 | -0.49 | 53.5 | 2.0 |
| mutton snapper | core | STX | MS-STX-01 | 2010 | 660 | 1995.0 | 16 | -1.34 | 82.4 | 2.1 |
| mutton snapper | core | STX | MS-STX-02 | 2018 | 681 | 2005.0 | 14 | -1.64 | 59.7 | 2.2 |
