# Supplementary figures and images for: Radiocarbon in otoliths of tropical marine fishes: Reference Δ14C chronology for north Caribbean waters
Source: PLoS One. 2021 May 12;16(5):e0251442. doi: 10.1371/journal.pone.0251442 (PMC8115809; doi:10.1371/journal.pone.0251442)

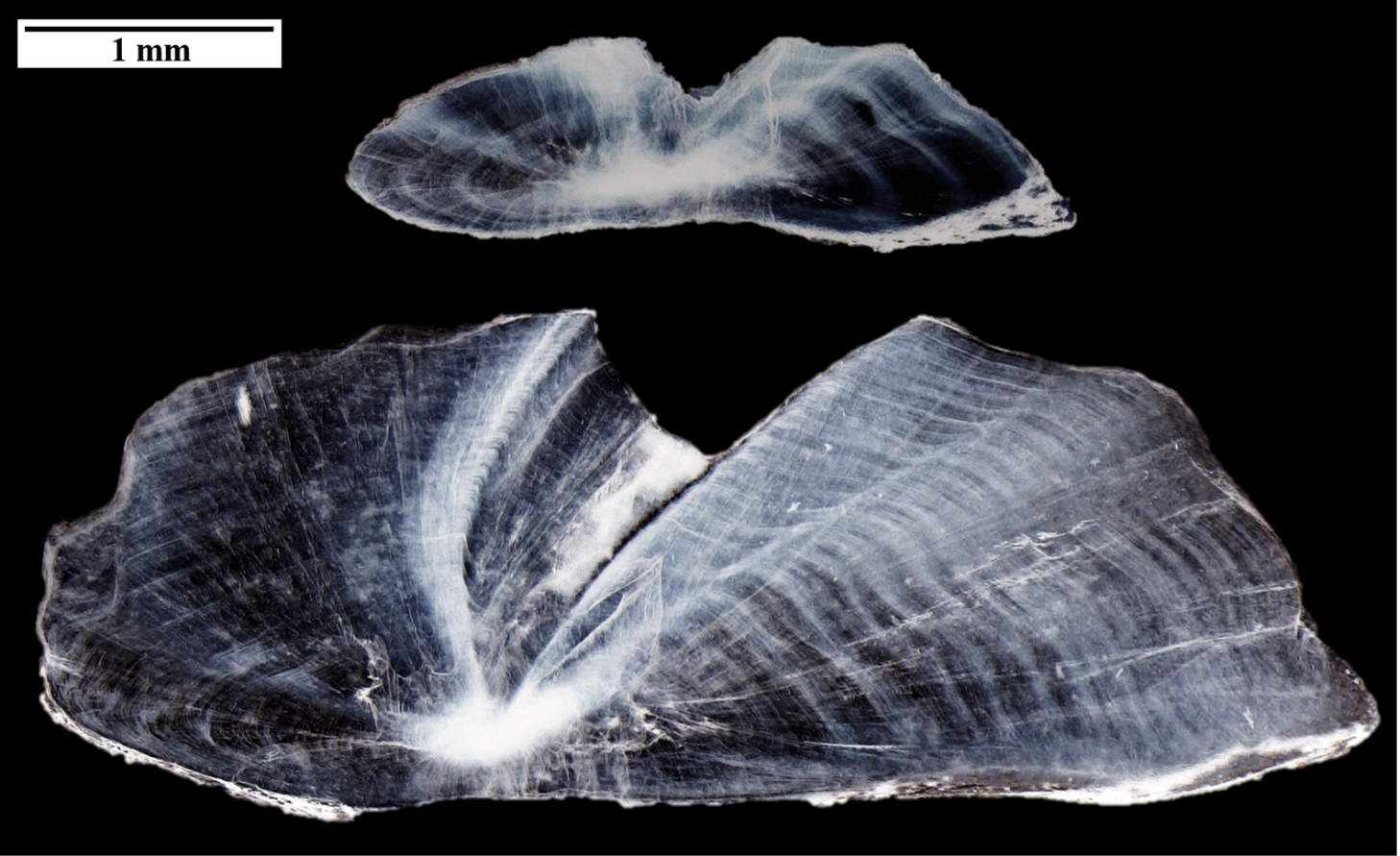

Supplement: S1 Fig — The top otolith section is from a 3 year old red hind and the bottom otolith section is from a 17 year old red hind. (TIF) [file pone.0251442.s002.tif]
